# Activation of the RhoB Signaling Pathway by Thyroid Hormone Receptor β in Thyroid Cancer Cells

**DOI:** 10.1371/journal.pone.0116252

**Published:** 2014-12-30

**Authors:** Sayaka Ichijo, Fumihiko Furuya, Hiroki Shimura, Yoshitaka Hayashi, Kazuya Takahashi, Kazuyasu Ohta, Tetsuro Kobayashi, Kenichiro Kitamura

**Affiliations:** 1 Third Department of Internal Medicine, Interdisciplinary Graduate School of Medicine and Engineering, University of Yamanashi, Yamanashi, Japan; 2 Department of Laboratory Medicine, Fukushima Medical University, Fukushima, Japan; 3 Department of Endocrinology and Metabolism, Division of Molecular and Cellular Adaptation, Research Institute of Environmental Medicine, Nagoya University, Nagoya, Japan; Nihon University School of Medicine, Japan

## Abstract

Thyroid hormone receptor (TR) mediates the crucial effects of the thyroid hormone (T3) on cellular growth, development, and differentiation. Decreased expression or inactivating somatic mutations of TRs have been found in human cancers of the liver, breast, lung, and thyroid. The mechanisms of TR-associated carcinogenesis are still not clear. To establish the function of TRβ in thyroid cancer cell proliferation, we constructed a recombinant adenovirus vector, AdTRβ, which expresses human TRβ1 cDNA. Thyroid cancer cell lines in which TRβ protein levels were significantly decreased as compared to intact thyroid tissues were infected with AdTRβ and the function of TRβ on cell proliferation and migration was analyzed. Ligand-bound TRβ induced HDAC1 and HDAC3 dissociation from, and histone acetylation associated with the RhoB promoter and enhanced the expression of RhoB mRNA and protein. In AdTRβ-infected cells, T3 and farnesyl transferase inhibitor (FTI)-treatment induced the distribution of RhoB on the cell membrane and enhanced the abundance of active GTP-bound RhoB. This RhoB protein led to p21-associated cell-cycle arrest in the G0/G1 phase, following inhibition of cell proliferation and invasion. Conversely, lowering cellular RhoB by small interfering RNA knockdown in AdTRβ-infected cells led to downregulation of p21 and inhibited cell-cycle arrest. The growth of BHP18-21v tumor xenografts *in* *vivo* was significantly inhibited by AdTRβ injection with FTIs-treatment, as compared to control virus-injected tumors. This novel signaling pathway triggered by ligand-bound TRβ provides insight into possible mechanisms of proliferation and invasion of thyroid cancer and may provide new therapeutic targets for thyroid cancers.

## Introduction

Thyroid hormone receptors (TRs) are ligand-dependent transcription factors that mediate the actions of the thyroid hormone (T3) in cellular development, growth and differentiation. Two human TR genes, THRA and THRB that are located on different chromosomes, encode T3-binding isoforms (TRα1, β1, β2, and β3) that are expressed in a tissue- and development-dependent manner [1]. Over the past decades, significant advances have been made in the understanding of TR actions in maintaining normal cellular function. However, the roles of TRs in human cancer are not well understood. The reduced expression of TRs because of hypermethylation or deletion of TR genes in human cancers suggests that TRs could function as tumor suppressors [2]. A close association of somatic mutations of TRs with thyroid cancers further supports the notion that the loss of normal functions of TR could lead to uncontrolled growth and loss of cell differentiation [3].

To understand the functional consequences of ligand-bound TRβ effects on downstream signaling pathways in thyroid cancer cells, we focused on RhoB that is a member of the Ras superfamily of isoprenylated small GTPases, which regulate actin stress fibers and vesicle transport [4]. Other RhoGTPases, which include RhoA and RhoC, promote oncogenesis, invasion, and metastasis [5]. In contrast, RhoB has antiproliferative and proapoptotic effects in cancer cells, and overexpression of RhoB can inhibit cell migration, invasion, and metastasis [6]. Membrane association of RhoB protein occurs through either geranylgeranylated (RhoB-GG) or farnesylated modifications. RhoB responds to farnesyl transferase inhibitor (FTI)-treatment by a gain-of-function mechanism that is characterized by elevation of the RhoB-GG form that inhibits proliferation or apoptosis of cancer cells [7]. Thus, altered expression and activity of RhoB may be crucial for cancer progression and therapeutic responses.

In the present study, we explored the function of ligand-bound TRβ in thyroid cancer cells. Ligand-bound TRβ induced RhoB protein expression, leading to increased expression of p21 followed by decreased cell proliferation and motility. FTI-treatment enhanced these antiproliferative functions of ligand-bound TRβ. Our results identify RhoB upregulation as a key step for targeting thyroid cancer cell proliferation and tumor progression. This novel signaling pathway triggered by ligand-bound TRβ provides insight into possible proliferation and invasion mechanisms of thyroid cancer.

## Materials and Methods

### Cell culture

BHP18-21 cells, which were reported by Ohta *et al.* [8], were provided as a gift by Dr. Jerome Hershman, UCLA. BHP18-21v cells, that express Pax-8, but do not express either the thyroglobulin or the thyroid transcription factor-1 gene, were isolated from BHP18-21v cells [9]. FRO and WRO cells, which were reported in citation [10], [11] were kindly provided by Dr. Shunichi Yamashita, University of Nagasaki. These cell lines are not *de* *novo* cell lines but have already been reported [8], [10], [11] and were kindly provided as gifts by our collaborators. All cells were grown in RPMI 1640 medium with 10% (v/v) fetal bovine serum (FBS) in a humidified incubator under a 5% CO_2_ atmosphere. DNA profiling of cancer cell lines was analyzed by Promega Japan (Tokyo, Japan) and is shown in Table 1. Thyroid hormone, triiodothyronine (T3) and the farnesyl transferase inhibitor (FTI), FTI-277, were purchased from Sigma Aldrich (St. Louis, MO). The cells were incubated with resin-stripped (T_3_-depleted) FBS [12] and were then infected with 30 MOI of AdTRβ or control AdLacZ. The cells were incubated in medium with or without 30 nM of T3 or 5 µM of FTI.

**Table 1:** DNA profiling of thyroid cancer cell lines.

Cell line	D3S1358	D8S1179	D21S11	D18S51	D5S818	D13S317	D7S820	D16S539	vWA	TH01	AM	TPOX	FGA
BHP18-21v	16,17	11,17	30,31.2	13,16	8,10	11,12	11	9	14,18	9	X	11	20,23
FRO	14,16	14	29,31	17	11,12	13	11	11,12	17,18	6	X	8,11	20
WRO	14,15	11,16	29	13	12	9,10	8,12	11,12	16,19	7,9.3	X	8	23

### Construction of the recombinant adenoviral vectors

AdTRβ is a ΔE1–ΔE3 recombinant adenovirus that expresses the human TRβ gene [13] under the control of the immediate early promoter of cytomegalovirus (CMV). Construction of the AdTRβ virus has been previously described [13]. AdLacZ, which contains the CMV promoter-controlled *lacZ* gene, was purchased from Quantum Biotechnologies (Montréal, Canada) and was used as a control. AdTRβPV is a recombinant adenoviral vector that expresses human TRβPV, which is a dominant negative mutant of TRβ [14], under the control of the cytomegalovirus immediate early promoter. The FLAG-TRβPV plasmid [15] was used as the template for cloning *TRβPV* into pShuttle2 (Clontech, Mountain View, CA) using the polymerase chain reaction. The PCR primers were: FLAG-ApaI-5′ (AAGGGCCCGCCGCCATGGACTACAAAGACGATGACGAC), and TRβPV-3′ KpnI (AATGGTACCTTCAGTCTAATCCTCGAACACTTCC) in which the underlines indicate restriction sites. An adenovirus vector was then constructed using the Adeno X Expressing System (Clontech) according to the manufacturer's protocol. Recombinant adenoviruses were plaque-purified, harvested 48 h after infection of 293 cells, and purified by double cesium chloride gradient ultracentrifugation [16]. Viral titers were determined by plaque assays with cultured 293 cells [16] and an adenovirus titration kit (Clontech).

### Plasmid construction and luciferase assays

The RhoB reporter plasmid −726/+86 RhoB-Luc plasmid was kindly provided by Dr. Dimitris Kardassis (University of Crete Medical School) [17]. Transient co-transfections were carried out in triplicate. The Renilla luciferase plasmid was co-transfected for normalization. Plasmid transfections were performed in AdTRβ or control virus-infected thyroid cancer cells using Lipofectamine 2000 (life technologies, Grand Island, NY). Twenty-four hours after transfection, the cells were treated with or without T3 for an additional 24 h. Dual luciferase assays were carried out according to the manufacturer’s instructions (Promega, Madison, WI).

### ChIP assay

ChIP assays were performed using a simple ChIP enzymatic chromatin immunoprecipitation kit (Cell Signaling Technology, Danvers, MA). AdTRβ-infected cells (5×10^7^) that were treated with or without T3 were fixed with 1% formaldehyde and digested chromatin was immunoprecipitated with anti-histone 3 (positive control), normal rabbit IgG (negative control), anti-acetyl histone 3 (Lys 9), anti-histone deacetylase (HDAC)1 or anti-HDAC3 antibodies. Five hundred µl of diluted chromatin were immunoprecipitated with 5 µg of each antibody and protein G magnetic beads according to the manufacturer’s instructions. Quantitative real-time (RT)-PCR was performed using a SYBR green real-time PCR kit (Life Technologies). The primer sequences used for RT-PCR were: forward 5′-GCAGCAGCAGCGCAGACT-3′ and reverse 5′-ACTCGGCCTAGCTCTCTC-3′.

### Quantitative real-time reverse transcriptase PCR

Total RNA was extracted from cells by using an RNeasy mini kit (Qiagen, Valencia, CA) according to the manufacturer’s instructions. After quantification by spectrophotometry, 5 µg of total RNA was reverse transcribed to obtain cDNA by using 160 µM deoxynucleotide triphosphate, 50 ng of random hexamer primers and 200 units of SuperScript II according to the manufacturer’s recommendations (Life Technologies). TaqMan probes for human RhoB, TRβ, and 18S rRNA were purchased from Life Technologies. mRNA levels were determined by real-time RT-PCR as previously described by Furuya *et al.* [18].

### Western blot analysis

This study was approved by the ethics committee of the University of Yamanashi. Normal thyroid tissues from the opposite lobe of carcinomas were obtained by surgery after the patients gave written informed consent. The documents regarding informed consent, which were approved by the ethics committee, were kept in the university hospital storage cabinet. Normal tissue from 4 of the patients was pooled for the Western blotting experiment. Tissues were frozen in liquid nitrogen immediately after resection. Protein lysates of normal thyroid tissue and of cancer cell lines were prepared by using cell lysis buffer (Cell Signaling Technology) according to the manufacturer’s instructions. Membrane protein was purified from AdTRβ-infected BHP18-21v cells by using a membrane protein purification kit (GE healthcare Life Science, Pittsburgh, PA) according to the manufacturer’s instructions. Determination of protein abundance by Western blot analysis was performed as described [19] using the following primary antibodies (1∶500 dilution): anti-RhoB and anti-phosphorylated Rb (Cell Signaling Technology); anti- PPARγ, anti-p21, anti-tubulin, and anti-GAPDH (Santa Cruz Biotechnology, Dallas TX). Negative control siRNA and siRNA against RhoB were purchased from Dharmacon (Thermo Fisher, Leicestershire, UK). Western blot analysis of siRNA-transfected cells was performed as follows: Cancer cell lines (1.0–1.3×10^5^) infected with adenovirus (MOI 30) were incubated with T3 for 12 h and then 50 nm of siRNA was transfected into the cells by using Lipofectamine 2000 (Life Technologies) according to the manufacturer’s instructions. After 48 h, the cells were washed with phosphate-buffered saline (Ca^2+^/Mg^2+^-free) and analyzed as described above.

### Immunoprecipitation analysis

To analyze the protein expression of TRβ in cells, 500 µg of protein lysate from thyroid tissue, HepG2 or virus-transfected thyroid cancer cell lines was immunoprecipitated with 5 µg of the C3 mouse monoclonal antibody against the C terminus of TRβ (Santa Cruz Biotechnology) or with 5 µg of mouse IgG as indicated in the text by using the Dynabeads protein G immunoprecipitation kit (Life Technologies) according to the manufacturer's protocol. Western blot analysis was performed using an anti-TRβ antibody (Santa Cruz Biotechnology) against an N-terminal TRβ peptide or an anti-TRα antibody (Santa Cruz Biotechnology) against an N-terminal TRα peptide. The cell lysates of AdTRβ-infected cells were immunoprecipitated with Rhotekin RBD tagged agarose beads and the abundance of GTP-bound RhoB was analyzed by pull-down assay, by using RhoB activation assay kit (Cell Biolabs, Inc. San Diego, CA).

### Cell-cycle analysis

The cells were stained with 20 µg/ml of propidium iodide (Life Technologies) and were analyzed using a BD Biosciences FACS Calibur flow cytometer (Franklin Lakes, NJ) and Cell Quest software version 3.0. The percentage of cells in the G_1_, S, or G_2_/M phase was calculated by using ModFitLT version 3.1.

### Cell proliferation and invasion assays

The number of cells was measured by using a nonradioactive cell proliferation assay (Cell Counting Kit-8; Dojindo, Kumamoto, Japan) as previously described by Furuya *et al* [19]. Cellular invasion was measured using the Cytoselect Cell Invasion assay kit (Cell Biolabs, Inc.), according to the manufacturer's instructions. Cell culture medium with 10% FBS was used as a chemoattractant in the lower well of the chamber. After rehydration of the basement membrane, thyroid cancer cell lines were seeded in the upper compartment of the chamber in serum-free medium (1×10^4^ cells per well) with T3 or FTI-treatment. After incubation at 37°C in 5% CO_2_ for 24 h, the non-invading cells were removed from the upper surface, and the cells that had invaded the membrane (8 µm pore size) to reach the lower surface were stained with CyQuant GR Fluorescent Dye. Their fluorescence was analyzed at 480 nm using a fluorescence plate reader.

### Xenografts of BHP18-21v cells and vector injection

BHP18-21v cells (1×10^7^) were transplanted into the abdominal subcutaneous tissue of 8-week-old male nude mice (BALB/C *nu/nu*). Three weeks later, when the tumors had reached approximately 1 cm in diameter, mice were randomly assigned to the experimental or control group (6 mice per group). Five×10^8^ plaque-forming units of AdTRβ or AdLacZ in 100 µl PBS were injected into the tumors. Adenovirus administration was repeated every 7 days. At each time point, all mice were also intraperitoneally injected with 100 mg/kg/body weight of FTI-277. On day 30, mice were euthanized by CO_2_ inhalation. Tumors which were removed from the mice were fixed in 10% buffered formalin and embedded in paraffin. Sections were permeabilized with 0.2% Triton X-100 in PBS for 10 min at room temperature. Serum free T3 and TSH levels were determined by using the free T3 ELISA kit (Phoenix Pharmaceuticals, Inc, CA) and a TSH assay kit (Uscn Life Science Inc, Wuhan, China), respectively, according to the manufacturers’ instructions. The institutional biosafety committee of the University of Yamanashi approved the animal procedures and the use of recombinant DNA.

### Statistics

Data are expressed as means ± S.D. Statistical analysis was performed using one-way analysis of variance or the unpaired 2-tailed Student's *t*-test, and probability values of less than 0.05 were considered to be statistically significant.

## Results

**Figure 1:**
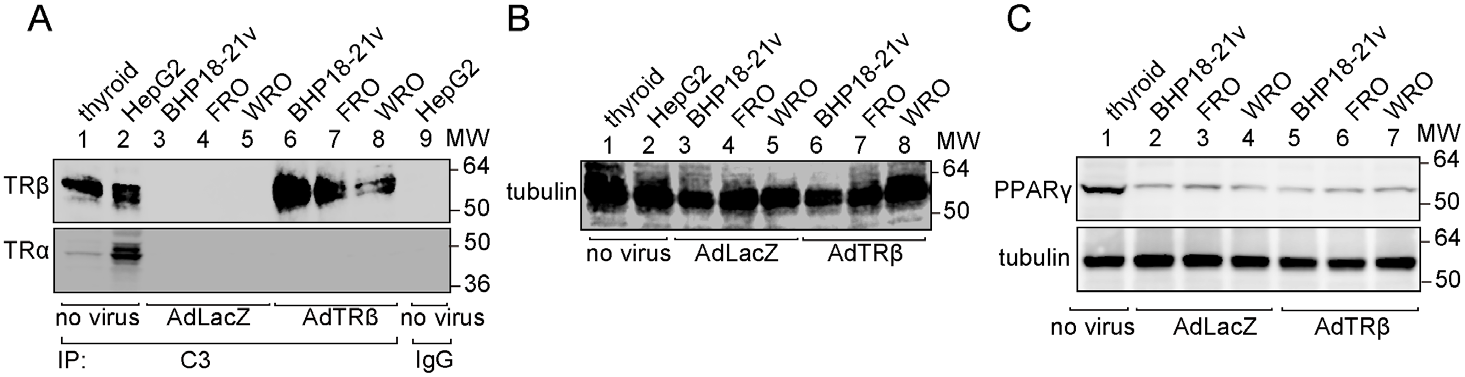
TRβ expression in intact thyroid tissues and thyroid cancer cell lines. A. Western blot of cell lysates (500 µg) prepared from normal thyroid tissue, from positive control HepG2 cells, or from the thyroid cancer cell lines BHP18-21v, FRO and WRO that were infected with AdTRβ or control AdLacZ virus. Lysates were immunoprecipitated (IP) with 5 µg of the mouse monoclonal anti-TRβ antibody (C3) that recognizes the TRβ C-terminus, or with 5 µg of normal mouse IgG (IgG) as indicated. Blots were probed with an antibody against an N-terminal TRβ peptide (TRβ) or with an antibody against an N-terminal TRα peptide (TRα). B. Tubulin expression in total cell lysates was used as a loading control. C. Western blotting analysis of the expression level of the PPARγ protein (upper panel) in cell extracts (20 µg of protein lysate). Tubulin was also blotted as a loading control (bottom panel).

To explore TRβ function in thyroid cancer cell proliferation, we focused on analysis of RhoB, which is a target of TRβ and is expressed at very low levels in 130 human cancer cell lines [20]. For the present study, we used the 3 thyroid cancer cell lines, BHP18-21v, FRO and WRO, which were infected with 30 MOI of AdTRβ or AdLacZ. We first analyzed TRβ protein expression in these cells. Five hundred µg of whole cell lysate protein was immunoprecipitated with a monoclonal antibody that recognizes the TR C-terminal region, followed by Western blot analysis with an antibody against an N-terminal TRβ peptide. As shown in Fig. 1A, TRβ expression was clearly observed in the positive controls; intact thyroid tissue (lane 1) and HepG2 cells that express functional TR [21] (lane 2), and also in AdTRβ-infected BHP18-21v, FRO and WRO cells (lanes 6–8). No TRβ protein expression was observed in AdLacz-infected BHP18-21v, FRO or WRO cells (lanes 3–5) or in HepG2 cells that were immunoprecipitated with a mouse monoclonal IgG antibody as a negative control (lane 9). Tubulin expression in the protein lysate (10 µg) was also analyzed as a loading control (Fig. 1B). We also analyzed TRα expression in these cell lysates by Western blot analysis of the immunoprecipitated proteins with an antibody against an N-terminal TRα peptide. TRα expression was observed in the positive controls; thyroid tissue and HepG2 (lanes 1 and 2, respectively). No TRα protein expression was observed in BHP18-21v, FRO or WRO cells that were infected with AdLacZ (lanes 3–5) or AdTRβ (lanes 6–8). Down-regulation of PPARγ expression and its activity is considered to be one mechanism of thyroid carcinogenesis [22]. We therefore also analyzed the protein expression of PPARγ in BHP18-21v, FRO or WRO cells (Fig. 1C). PPARγ expression was decreased in these cancer cells compared with that of intact thyroid tissue but infection with AdTRβ had no effect on the expression of PPARγ in thyroid cancer cells. These data indicate that the three AdTRβ or AdLacZ infected cell lines tested are a good model for analysis of TRβ function.

**Figure 2:**
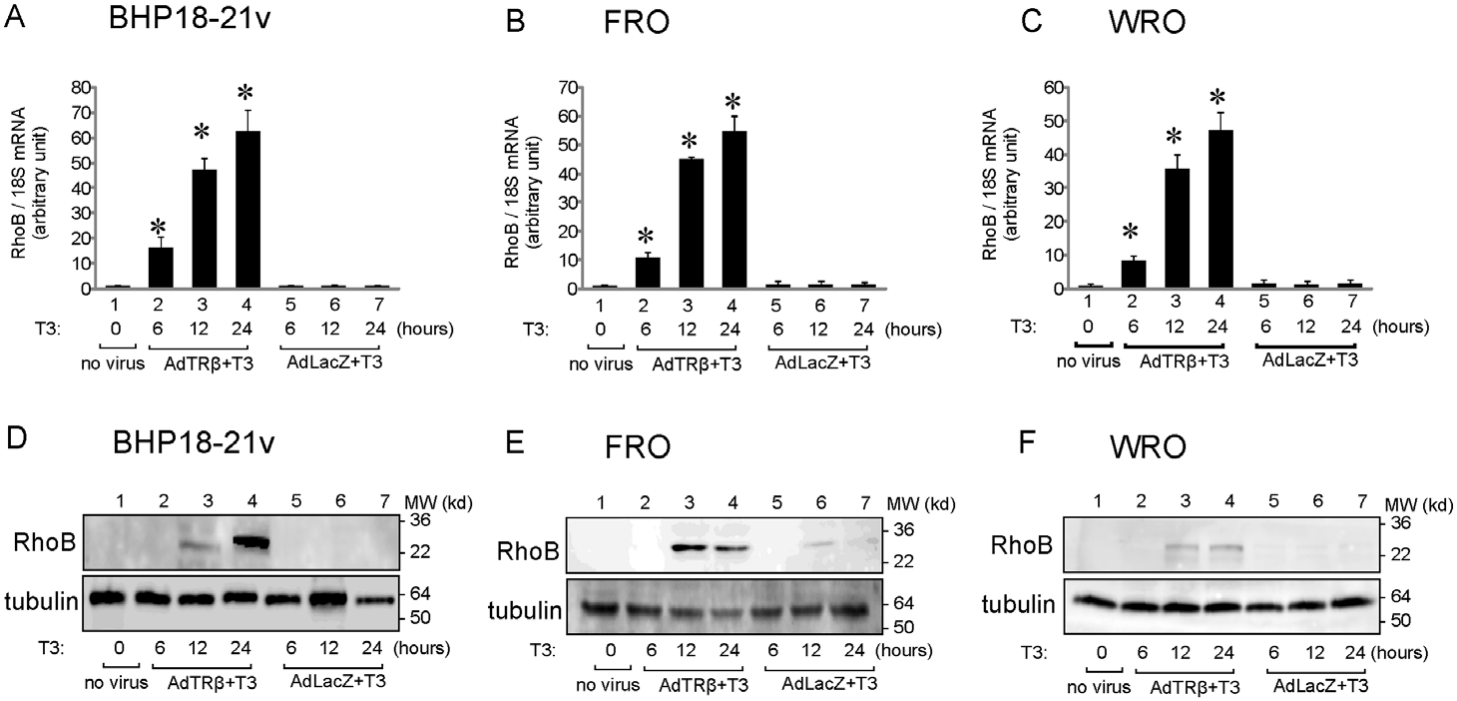
Expression of RhoB in AdTRβ-infected thyroid cancer cells. Expression levels of RhoB mRNA (A–C) or protein (D–F) in AdTRβ-infected, control AdLacZ-infected, or non-infected thyroid cancer cell lines exposed to 30 nM of T3 for 0, 6, 12 or 24 h as indicated. (A–C)The expression of RhoB and 18S mRNA was determined using real-time RT-PCR with 100 ng of cDNA. Relative mRNA expression levels were determined by arbitrarily setting the value for control virus-infected cells incubated in T3-free medium to 1. Data are expressed as means ± S.D. (*n*  = 6). *, *p*<0.05. (D–F) Western blot analysis of 20 µg of protein lysates of the cells was performed using antibodies against RhoB (upper panel) or tubulin (lower panel), which was used as a loading control.

We next analyzed the effect of T3 treatment of AdTRβ- or AdLacZ-infected thyroid cancer cell lines on RhoB expression (Fig. 2A–C). T3-treatment (30 nM) of AdTRβ-infected (lanes 1–4) but not of AdLacZ-infected (lanes 5–7) BHP18-21v, FRO and WRO cells for 6, 12 or 24 h significantly enhanced the expression of RhoB mRNA in a time-dependent manner compared to non-treated non-virally infected cells. Furthermore, Western blotting analysis indicated that T3-treatment for 12 or 24 h induced RhoB protein expression in AdTRβ-infected BHP18-21v, FRO and WRO cells but not in AdLacZ-infected cells (Fig. 2D–F, lanes 2–4 and lanes 5–7 respectively). Thus, induction of RhoB mRNA and protein expression are T3/AdTRβ-dependent in thyroid cancer cell lines.

**Figure 3:**
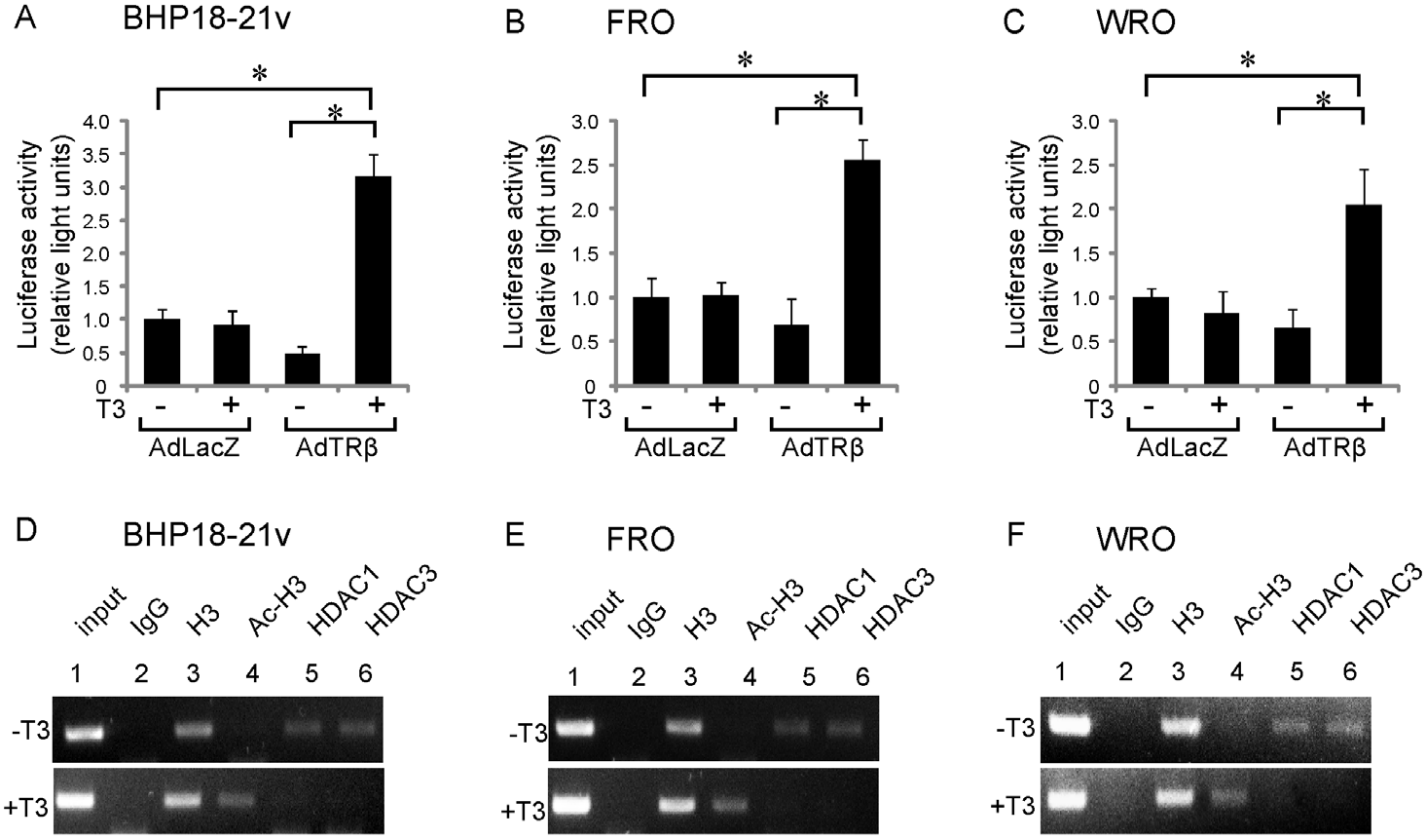
Ligand-bound TRβ binds the RhoB promoter and upregulates transcriptional activation. (A–C) RhoB promoter-Luciferase reporter assays. Adenovirus-infected BHP18-211v (A), FRO (B), or WRO (C) cells were transfected with 1 µg of a RhoB promoter-Luciferase reporter plasmid and were incubated for an additional 24 h in the absence or presence of 30 nM T3. Relative promoter activities (Luciferase activity) were determined by arbitrarily setting the value for control virus-infected cells incubated in T3-free medium to 1. Data are expressed as means ± S.D. (*n*  = 6). *, *p*<0.05. (D–F) ChIP assay using AdTRβ-infected BHP18-211v (D), FRO (E), or WRO (F) cells treated with or without 30 nM T3. Antibodies that recognize histone3 (H3), acetylated H3, HDAC1, HDAC3 or normal rabbit IgG were used for immunoprecipitation of the chromatin. Input indicates 10% of the chromatin. PCR products were separated on a 2% of agarose gel.

To clarify the mechanisms through which ligand-bound TRβ enhanced the expression of RhoB in AdTRβ-infected BHP18-21v, FRO and WRO cells, we analyzed the effect of T3 treatment of these cell lines on the transcriptional activity of the RhoB promoter. AdTRβ- or control AdLacZ-infected cells were therefore transiently transfected with the RhoB promoter-luciferase reporter construct, −726/+86 RhoB-Luc. Luciferase activity driven by the RhoB promoter was significantly enhanced in AdTRβ-infected cells, but not in AdLacZ-infected cells following T3-treatment (Fig. 3A–C).

Transcription of the RhoB promoter is inhibited by transcriptional repressors that recruit histone deacetylases (HDACs) to remove the acetylation of lysine residues on histones3 (H3) tails [23]. It is well established that the acetylation status of the tail domains of histones3 (H3) change during transcriptional activation of the RhoB promoter [24]. To determine if induction of RhoB gene expression by ligand-bound TRβ correlates with changes in the acetylation status of H3 in the promoter region of the RhoB gene, chromatin immunoprecipitation (ChIP) assays were performed. AdTRβ-infected BHP18-21v, FRO or WRO cells were incubated with or without T3 for 24 h and ChIP assays were performed using antibodies to H3, acetylated H3, HDAC1 and HDAC3. As shown in Fig. 3D–F, H3 was found in fragments of the RhoB promoter (lane 3) in AdTRβ-infected cells with or without T3-treatment. However, HDAC1 and HDAC3 were associated with fragments of the RhoB promoter only when the cells were incubated without T3, and not when the cells were incubated with T3 (HDAC1/3; lanes 5 and 6, respectively). Consistent with these results, no acetylated H3 was associated with the promoter in the absence of T3 treatment, whereas in the presence of T3, the H3 that was associated with the promoter was highly acetylated (lane 4). These results indicated that ligand-bound TRβ induced RhoB transcription via alteration of the histone acetylation status of thyroid cancer cells.

**Figure 4:**
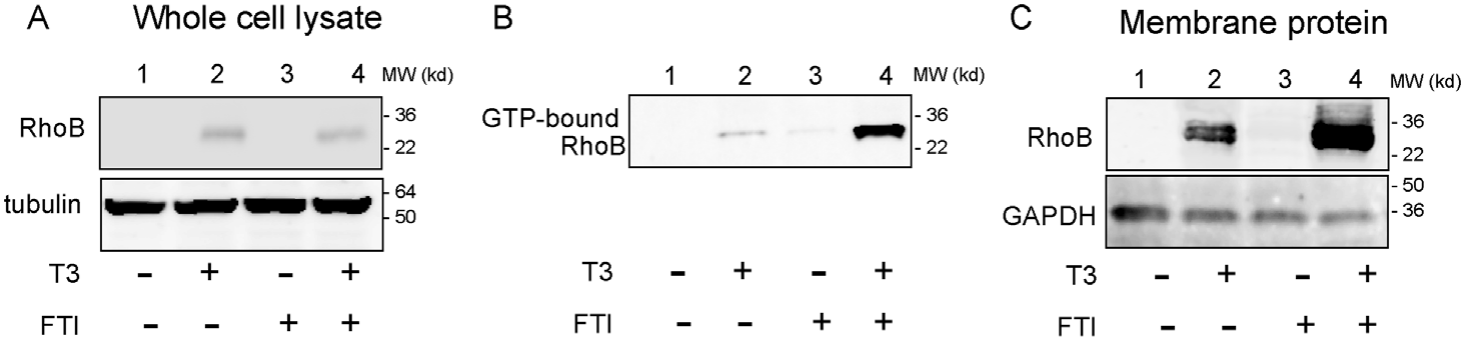
T3 and farnesyl transferase inhibitor (FTI)-treatment regulated RhoB activity and cellular distribution. AdTRβ-infected BHP18-211v cells were exposed to30 **nM T3** and/or 5 µM **of** the farnesyl transferase inhibitor (FTI) for 24 h and were then analyzed as follows. A. Western blotting analysis of the expression level of the RhoB protein (top panel) in cell extracts (20 µg of protein lysate). Tubulin was also blotted as a loading control (bottom panel). B. Cell lysates were immunoprecipitated with Rhotekin RBD-tagged agarose beads. GTP-bound RhoB in the precipitates was analyzed by Western blotting using an antibody against RhoB. C. Western blotting analysis of RhoB protein in the membrane protein fraction. GAPDH was blotted as a membrane protein marker loading control (bottom panel).

We next analyzed the effect of ligand-bound TRβ on the cellular localization and function of RhoB. For this purpose we employed the agent FTI. FTI treatment of cells induces the membrane association of RhoB by modification of RhoB through the addition of lipid moieties [4]. Membrane association of RhoB is important for its activity. We first determined the effect of 5 µM of FTI treatment on RhoB protein expression in AdTRβ-infected BHP18-21v cells that were treated with or without T3 (30 nM). Western blotting of whole cell lysates of treated/untreated AdTRβ-infected BHP18-21v cells with RhoB antibody (Fig. 4A) indicated that FTI-treatment did not alter T3 induction of RhoB expression (lanes 2 and 4) and did not enhance RhoB protein expression in AdTRβ-infected BHP18-21v cells in the absence of T3-treatment (lane 3).

We then analyzed the effect of T3 treatment with/without FTI co-treatment on RhoB activity. Like other small GTPases, RhoB regulates molecular events by cycling between an inactive GDP-bound form and an active GTP-bound form. In the active GTP-bound form, RhoB specifically binds to the Rho-binding domain (RBD) of Rhotekin to regulate downstream signaling cascades. We therefore analyzed the abundance of GTP-bound RhoB in cell lysates of AdTRβ-infected BHP18-21v cells that were treated with or without T3 and/or FTI by immunoprecipitation of cell lysates with Rhotekin RBD-tagged agarose beads followed by immunoblotting for RhoB (Fig. 4B). These pull-down assays indicated that not only the abundance of RhoB but also the abundance of the active GTP-bound form of RhoB in AdTRβ-infected BHP18-21v cells was enhanced by T3-treatment (lane 2) and strongly enhanced by FTI and T3 co-treatment (lane 4). No GTP-bound RhoB was detected in FTI-treated cells without T3-treatment (lane 3). These results indicated that the ligand-bound AdTRβ-induced RhoB protein in BHP18-21v cells was an active kinase whose activity was enhanced by co-treatment with FTI.

To determine if T3 and FTI could induce membrane association of RhoB in AdTRβ-infected BHP18-21v cells, cell surface proteins were biotinylated. Biotinylated proteins in cell lysates were then pulled down with streptavidin beads and analyzed by immunoblotting with an anti-RhoB antibody (Fig. 4C). T3-treatment induced the expression of RhoB at the cell membrane (lane 2), which was greatly enhanced by co-treatment with FTI (lane 4). FTI-treatment did not enhance RhoB protein expression at the cell membrane of AdTRβ-infected BHP18-21v cells in the absence of T3-treatment (lane 3). These results indicated that T3-induced membrane association of RhoB that was enhanced by FTI co-treatment and were consistent with T3 induction of RhoB activity in AdTRβ-infected BHP18-21v cells.

**Figure 5:**
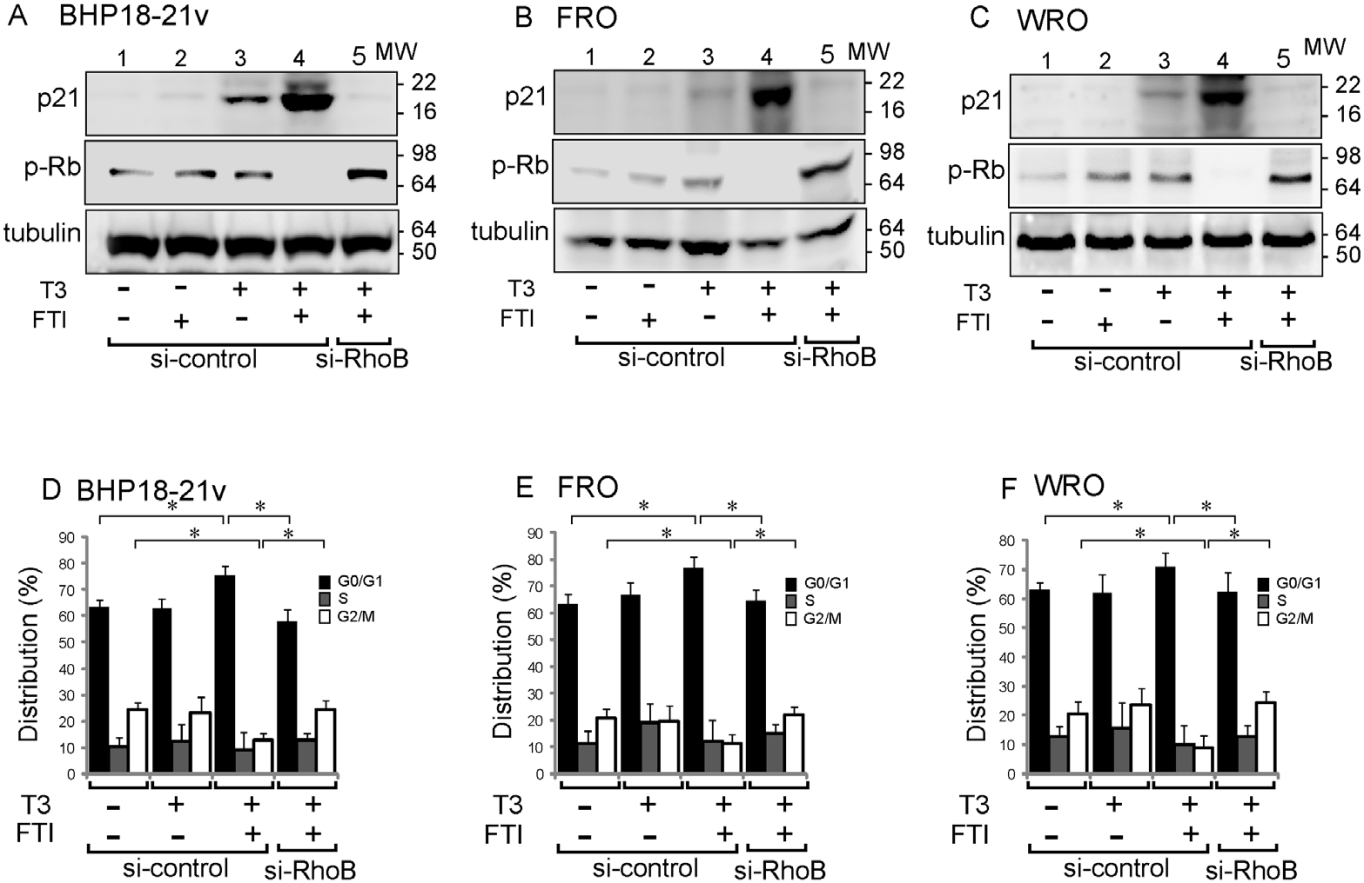
TRβ infection–induced p21 expression and Go/G1 growth arrest. (A–C) Western blot analysis of the expression level of p21 or of the phosphorylated Rb protein in AdTRβ-infected BHP18-21v (A), FRO (B), or WRO (C) cells that were exposed to 30 nM of T3 alone for 12 h or to 30 nM T3 plus 5 µM of FTI for 12 h, with or without siRNA knockdown of RhoB as indicated. Tubulin was blotted as a loading control (bottom panels). (D–F) The percentage of cells in the G_0_/G_1_, S, or G_2_/M phase was calculated by using ModFitLT version 3.1. Data are expressed as means ± S.D. (*n*  = 6). *, *p*<0.05.

The cyclin-dependent kinase (CDK) inhibitor, p21, is a negative regulator of cell-cycle progression and is one of the downstream targets of RhoB. To confirm the activity of RhoB in AdTRβ-infected BHP18-21v cells treated with T3 and/or FTI, we analyzed the protein expression of p21 by Western blot analysis (Fig. 5A–C). p21 expression was analyzed in cells transfected with RhoB-targeted or control siRNA to confirm the RhoB dependence of p21changes. Three thyroid cancer cells were pre-cultured in T3-free medium with stripped serum, as described in “Materials and Methods”. FTI-treatment did not enhance p21 protein expression in siRNA-control AdTRβ-infected cells without T3-treatment (lane 2). In AdTRβ-infected cells, the expression level of p21 was increased in cells treated with T3 (lane 3) and was greatly enhanced by co-treatment with FTI (lane 4) compared to non-treated cells (lane 1). However, p21 was not induced by T3 plus FTI treatment of AdTRβ-infected cells 24 h after transfection with RhoB-targeted siRNA (lane 5). p21 inhibits the activities of CDKs, leading to hypo-phosphorylation of retinoblastoma protein (Rb), which in turn causes cell-cycle arrest at the G0/G1 phase [25]. We analyzed the phosphorylation of Rb (p-Rb) in AdTRβ-infected thyroid cancer cells treated with T3 and/or FTI [(Fig. 5 (A–C)). Rb was phosphorylated in AdTRβ-infected BHP18-21v, FRO or WRO cells with or without T3-treatment (lanes 1 and 3). FTI-treatment alone did not inhibit the phosphorylation of Rb in AdTRβ-infected cells (lane 2). Rb phosphorylation was significantly decreased in AdTRβ-infected cells by co-treatment with T3 and FTI (lane 4). On the other hand, inhibition of Rb phosphorylation was not observed by T3 plus FTI treatment of AdTRβ-infected cells after transfection with RhoB-targeted siRNA (lane 5).

We further studied the cell cycle stages of these cells using flow cytometry (Fig. 5D–F). In si-control AdTRβ-infected cells, treatment with T3 plus FTI induced a significant increase in the G0/G1 phase population compared to non-treated or T3-treated cells, whereas in RhoB-targeted siRNA-transfected cells, T3+ FTI treatment had no effect on the cell population in the G0/G1 phase. Furthermore, treatment with T3 plus FTI reduced the number of cells in the G2/M phase in si-control- but not in RhoB-targeted siRNA-AdTRβ-infected cells. These results indicate that the enhanced RhoB expression in AdTRβ-infected cancer cells that were treated with T3 and FTI, was accompanied by increased expression of p21 and inhibition of cell-cycle progression.

**Figure 6:**
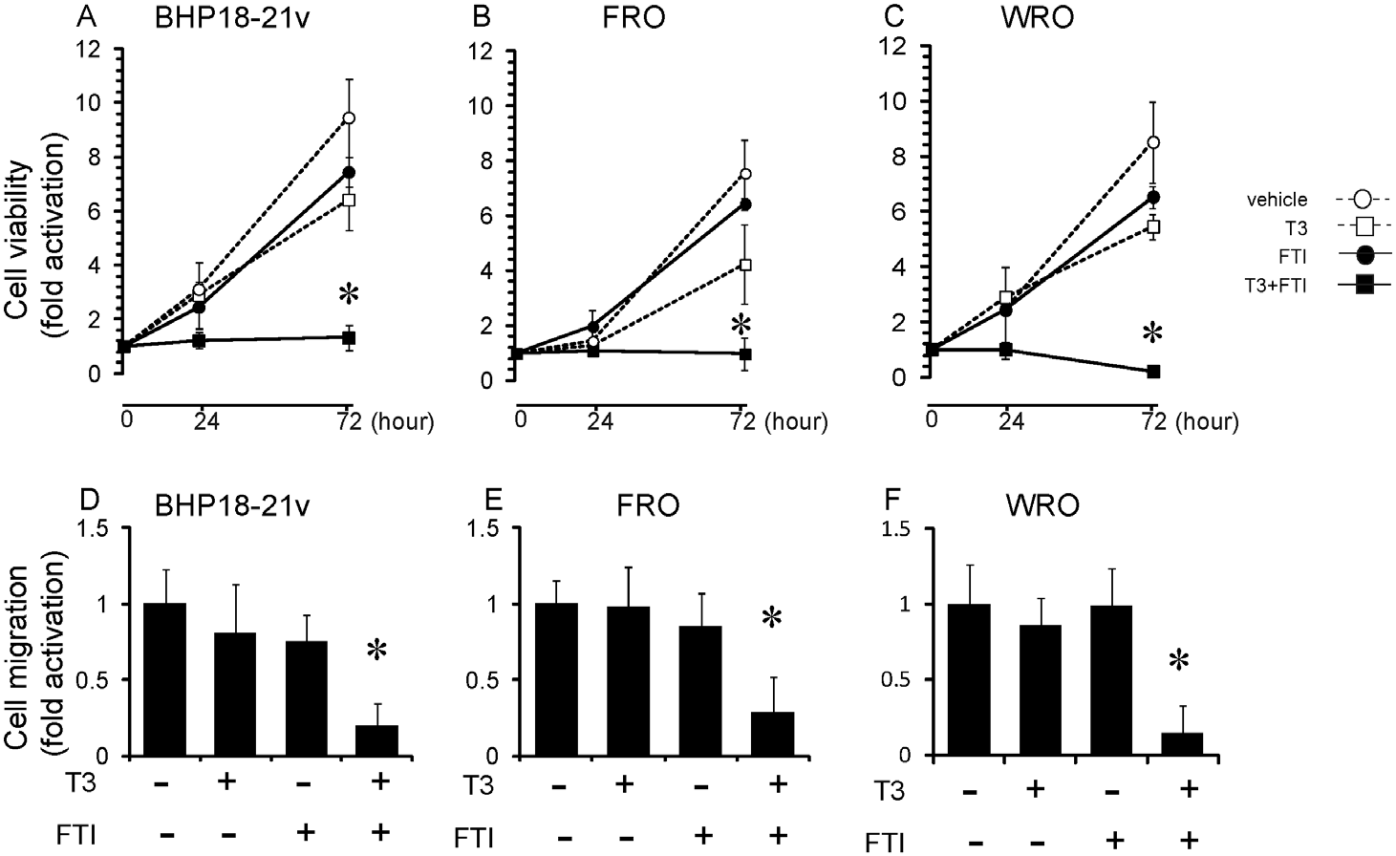
Effects of ligand-bound TRβ on cancer cell proliferation and migration. (A–C) Cell proliferation assay. BHP18-21v (A), FRO (B) or WRO (C) cells were incubated in T3-depleted medium for 24 h and were then infected with 30 MOI of AdTRβ. The cells were then incubated in medium with T3 and/or FTI for an additional 24 or 72 h. Relative cell numbers were determined by arbitrarily setting the value for control cultures incubated in T3-free medium to 1. Data are expressed as means ± S.D. (*n*  = 6). *, *p*<0.05. (D–F) AdTRβ-infected BHP18-21v (D), FRO (E) or WRO (C) cells were seeded in the upper compartment of a migration chamber. After 24 h incubation, cells that had migrated to the lower surface were stained and quantified using fluorescence signals. Relative signals were determined by arbitrarily setting the value for control cultures incubated in T3-free medium to 1. Data are expressed as means ± S.D. (*n*  = 6). *, *p*<0.05.

To confirm that the observed changes in p21 expression reflected changes in cell proliferation, we next analyzed the effects of TRβ on cancer cell proliferation using the MTT assay. For this assay, BHP18-21v, FRO or WRO cells were infected with 30 MOI of AdTRβ and, after 12 h of incubation in adenovirus-containing medium, the cells were cultured with or without T3 (30 nM) and 5 µM of FTI for 24 or 72 h. After 24 and 72 h of incubation cell number had increased in the absence of T3-treatment but was decreased in the presence of T3 and was significantly decreased in the presence of T3 plus FTI (Fig. 6A–C).

As a further measure of RhoB activity, we next analyzed cell invasion as RhoB has been reported to negatively affect cell migration. We analyzed the effect of T3 and FTI treatment on the invasive properties of AdTRβ transfected BHP18-21v, FRO or WRO cells using a cell invasion assay chamber. The cancer cells that invaded through the 8 µm pores of the dividing membrane to the lower chamber were quantified using fluorescence measurements. T3 plus FTI treatment significantly inhibited cell invasion of BHP18-21v, FRO and WRO cells compared the control group (80%, 71% and 85% reduction respectively; Fig. 6D–F). FTI treatment alone had no significant effect on migration. The combined results indicated that ligand-bound TRβ induced the expression of RhoB which acts as a critical mediator of the cellular response to FTI and inhibits thyroid cancer cell proliferation and migration.

**Figure 7:**
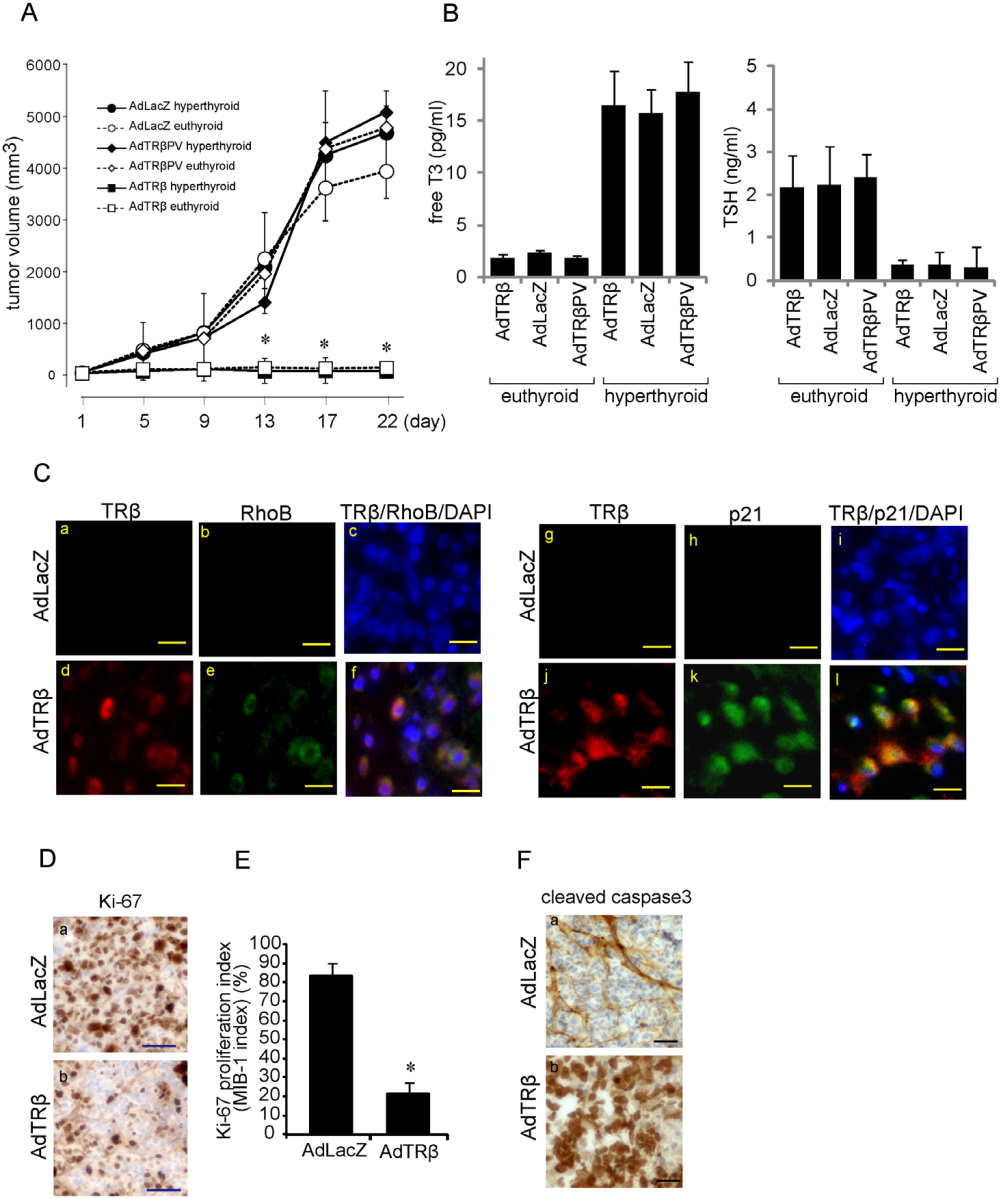
Effects of ligand-bound TRβ and FTI on BHP18-21v xenografts. A. Relative tumor volume of AdTRβ-, AdLacZ-, or AdTRβPV treated xenografts over 22 days following viral injection was calculated by the formula V = 1/2 (length×width^2^). All mice were intraperitoneally injected with 100 mg/kg/body weight of FTI at each time points of viral injection. Data are expressed as means ± S.D. (*n*  = 6). *, *p*<0.05. B. Serum free T3 and TSH levels of adenovirus-injected and FTI-treated mice were analyzed by ELISA. The data points represent means ± S.D. (*n*  = 6). C. RhoB and p21 levels in the tumors were visualized with anti-RhoB and anti-p21 antibodies, respectively, and Alexa Fluor 555-conjugated second antibody (red); transfected TRβ was visualized with an anti-TRβ antibody and an Alexa Fluor 488-conjugated secondary antibody (green). Scale bars, 10 µm. D. Ki-67-positive cells are indicated in AdLacZ (a)- or AdTRβ (b)- treated xenografts on day 30. Scale bars, 50 µm. E. The Ki-67 proliferation indices (MIB-1 indices) of the xenografts are shown as means ± S.D. (*n*  = 6). *, *p*<0.05. F. Cleaved caspase 3-positive cells are indicated in AdLacZ and FTI (a) or AdTRβ and FTI (b)- treated xenografts on day 30. Scale bars, 20 µm.

To evaluate the efficiency of AdTRβ in inhibition of cancer cell growth in vivo, 1×10^7^ BHP18-21v cells were injected into the left flanks of nude mice. The BHP18-21v cell line was selected for these experiments because these cells formed large tumors in nude mice, compared with FRO and WRO cell lines (data not shown). One week after injection, the cells formed small tumors at the injection sites. The tumors had grown to 1 cm in a diameter by 3 weeks after injection. At this point (day 1), as well as on days 7, 14 and 21, AdTRβ, AdLacZ, or AdTRβPV, which expresses a ligand-binding domain mutant of TRβ, was injected into the tumors (5×10^8^ PFU in 100 µl PBS). At the same time points all mice were intraperitoneally injected with 100 mg/kg/body weight of FTI. To analyze the effects of a hyperthyroid state, AdTRβ- or AdLacZ-infected mice were intraperitoneally injected with or without T3 (250 µg/kg/body weight) for 3 days. In the hyperthyroidism group, adenovirus injection into the tumors was repeated on days 7, 14 and 21 following T3-treatment. Tumor volumes were calculated at various time points from caliper measurements, as described in Materials and Methods. In AdTRβ-infected mice, there was a period of continued growth (5 days) followed by a decrease in tumor size. As shown in Fig. 7A, after 13 days the mean tumor volume in the AdTRβ-treated groups with or without T3-treatment was significantly less than that of the control group (*p*<0.01). The final tumor volumes of AdTRβ-, AdLacZ-, and AdTRβPV- treated mice with or without T3-treatment on day 22 were 136±30.1 vs. 142±41.7, 4898±265 vs. 5087±334, and 4786±727 vs. 5081±421 mm^3^, respectively. The final tumor volumes of AdTRβ-treated mice were significantly smaller than those of mice that were infected with AdLacZ or AdTRβPV. In AdTRβ-infected mice without T3 treatment, serum free T3 and TSH concentrations were 1.84±0.39 and 2.16±0.76, respectively (Fig. 7B). There were no differences in these values between AdLacZ- and AdTRβPV- treated mice. The free T3 or TSH levels of these mice were almost within the euthyroid range as indicated by a previous report [26]. In contrast, in T3-treated mice the concentration of free T3 was significantly elevated and serum TSH levels were suppressed compared with euthyroid mice. These results indicated that ligand-bound TRβ inhibits thyroid cancer cell growth and that the T3 level of euthyroid mice is sufficient for induction of TRβ-associated tumor growth suppression.

Immunohistochemical analysis indicated that ligand-bound TRβ induced the expression of RhoB in the cytoplasm (Fig. 7C) of AdTRβ-treated tumors in the euthyroid state, whereas RhoB expression was not observed in AdLacZ-treated tumors. Ligand-bound TRβ-induced p21 expression was clearly observed in the nucleus of AdTRβ-infected tumors, but p21 expression was not observed in AdLacZ-treated tumors. Immunohistochemical analysis of the proliferation marker Ki-67 in xenograft tissue sections showed that the fraction of proliferating cells is lower in AdTRβ-treated tumors versus AdLacZ-treated tumors (Fig. 7D; b and a respectively). Quantification of proliferation indicated that 14.0% ±4.2% and 92.0% ±4.8% of the cells were proliferating in AdTRβ- and AdLacZ-treated tumors, respectively (*p*<0.01) (Fig. 7E). Furthermore, cleaved caspase 3 expression was induced in AdTRβ-treated tumors but not in AdLacZ-treated tumors (Fig. 7F). Thus, TRβ overexpression reduced the proliferation and induced the apoptosis of human thyroid carcinoma cells xenografted in mice and strongly inhibited tumor growth.

## Discussion

The present study focused on the functional characterization of ligand-bound TRβ in human cancer cells. We provide evidence that ligand-bound TRβ activates the RhoB signaling pathway and inhibits cancer cell proliferation both in vitro and in vivo. Previous reports of poorly differentiated thyroid cancers in knock-in mice with a dominant-negative TRβ mutation strongly suggest the involvement of TRβ in carcinogenesis [2], [3], [27]. Our findings support the hypothesis that TRβ acts as a tumor suppressor in several types of cancer.

RhoB differs from the other GTPases RhoA and RhoC in that it is postulated to be a tumor suppressor because its expression is decreased in a number of tumor cell types [4]. Indeed, RhoB-null mice have increased susceptibility to skin tumor carcinogenesis [28]. Marlow et al. reported that reactivation of suppressed RhoB is a critical step for inhibition of the proliferation of anaplastic thyroid cancers [29]. In the current study, we analyzed 3 thyroid cancer cell lines, BHP18-21v; papillary thyroid carcinoma, FRO; anaplastic thyroid carcinoma, and WRO; follicular thyroid carcinoma [11] that had lost the expression of endogenous TR and in which RhoB protein expression was also not observed by Western blotting analysis. These findings suggested the possibility that endogenous TR, when present, might function as a tumor suppressor by inducing RhoB protein expression.

Indeed TR has been associated with transcription regulation of a number of genes via control of histone acetylation. Thus, unliganded TR binds with the nuclear receptor corepressor (NCoR) that recruits histone deacetylase (HDAC)-containing complexes to the promoter regions of genes [30]. These HDACs de-acetylate histones and repress promoter-activity [31], [32]. On the other hand, an increasing number of coactivators have been implicated in T3-dependent gene activation including SRC1 [33], CBP and p300 [34]. These coactivators have been shown to possess intrinsic histone acetyltransferase (HAT) activity. Acetylation of core histones in chromatin has long been proposed to facilitate transcription. Our results showing T3/TRβ dependent RhoB promoter transcription from a reporter plasmid, and T3/TRβ dependent regulation of Histone H3 acetylation on the RhoB promoter, are consistent with these data. Our data are also consistent with other reports regarding a role for HDACs in the regulation of RhoB expression. Thus, HDAC1 represses RhoB expression in lung cancer cells and treatment with the HDAC inhibitor, trapoxin A enhanced the transcription and expression of RhoB mRNAs [23]. The T3-dependent increased levels of RhoB mRNA, protein, and promoter activity observed in our study, coupled with the ChiP assay data, suggested that the RhoB gene was transcriptionally regulated by the T3-bound TRβ via HDAC inhibition. Our findings therefore establish RhoB as a direct transcriptional target of liganded-TRβ in thyroid cancer cell. Although previous reports indicated that FTI-treatment enhances the expression of RhoB in gastric cancer [35] or breast cancer [36] cells, in the present study, FTI-treatment did not enhance the expression of RhoB in BHP18-21v, FRO, or WRO cells. These differences may be caused by the HDAC status of the RhoB promoter region of these cancer cells.

In our study, T3-treatment of AdTRβ-infected BHP18-21v cells enhanced the expression of RhoB both in the cytoplasm and on the cell surface and enhanced the active GTP-bound form of RhoB. Furthermore, concomitant FTI-treatment greatly enhanced the expression of surface RhoB and of the active, GTP-bound form of RhoB. These data are consistent with the known localization of RhoB and the effect of FTI. Thus RhoB is known to be posttranslationally modified by the addition of lipid moieties (geranyl-geranyl or farnesyl groups), which enable RhoB to interact with components of the cell membrane [4]. RhoB localizes to both the plasma membrane and the membrane of endosomes [37] and has specific functions in endosomal trafficking. RhoB responds to FTI-treatment by a gain-of-function mechanism that is characterized by elevation of the geranyl-geranylated isoform of RhoB that inhibits the proliferation of cancer cells [4]. The combined data suggest that the liganded-TRβ-induced repression of cell proliferation that we observed might be mediated by localization of the induced RhoB to cellular membranes, from where it exerts its anti-proliferative activity.

Our results indicated that the ligand-bound TRβ signaling pathway led to tumor growth inhibition, not only *in* *vitro* but more importantly *in* *vivo* in a tumor xenograft model. Biochemical analysis *in* *vitro* suggested that this inhibition occurred via a RhoB-dependent mechanism that is upstream of p21. The p21 protein is a major player in cell-cycle control. Once activated, p21 exerts a negative effect on cell-cycle progression by preventing formation of the CDK2/cyclin E complex. Although p53 plays a key role in p21 regulation, induction of p21 expression can also occur in a p53-independent manner [38], [39]. The effect of liganded TRβ mediated-inhibition of cell-cycle progression and upregulation of RhoB and p21, occurred in cancer cells in which p53 function is disrupted [10]. Therefore, in our experimental model, p53 is unlikely to promote TRβ-induced p21 expression. Furthermore, when the activation of RhoB that was induced by T3/TRβ was inhibited by siRNA, the induction of p21 was not observed in these thyroid cancer cell lines. In AdTRβ-infected cancer cells treated with T3, ligand-bound TRβ enhanced the expression of p21, while Rb protein was still phosphorylated. On the other hand, FTI and T3 treatment of AdTRβ-infected cancer cells significantly enhanced the expression of p21 leading to hypo-phosphorylation of Rb, which in turn caused cell cycle arrest at the G0/G1 phase. Consistent with these findings, treatment of AdTRβ-infected cells with T3 alone did not enhance the number of cells in the G0/G1 phase whereas treatment with T3 and FTI did enhance the number of cells in the G0/G1 phase. Our results therefore identify RhoB upregulation as a key step for inhibition of thyroid cancer cell proliferation and therefore tumor progression via activation of p21.

The thyroid gland, which is a relatively common site for the development of malignant neoplasms, gives rise to 90% of all endocrine cancers. Thyroid cancer generally has a good prognosis. However, poorly differentiated or anaplastic thyroid carcinoma, which constitutes about 5%–14% of all thyroid carcinomas, is highly malignant and has a median survival time of only 2–6 months; anaplastic thyroid carcinoma rapidly invades adjacent structures and metastasizes throughout the body, especially to the lung [40], [41]. Since no effective therapy is available for these aggressive types of thyroid carcinoma, novel therapeutic strategies including gene therapy are urgently needed. Our findings indicate that the TRβ-induced pathways acted in concert to delay tumor progression and block metastatic spread. We therefore speculate that this TRβ-induced suppression of cancer cell growth may confer therapeutic effects on poorly differentiated and anaplastic thyroid carcinomas and that the underlying biochemical pathway may provide novel therapeutic targets for these cancers.
